# Caught in the Clot: A Case Report of Arrested Pulmonary Embolism

**DOI:** 10.7759/cureus.61213

**Published:** 2024-05-28

**Authors:** Dhruvkumar Thakkar, Varsha Shinde

**Affiliations:** 1 Emergency Medicine, Dr. D. Y. Patil Medical College, Hospital and Research Centre, Dr. D. Y. Patil Vidyapeeth (Deemed to be University), Pune, IND

**Keywords:** clot, embolism, thrombolysis, pe, pte, pulmonary embolism, pulseless electrical activity, cardiac arrest, thrombolytic therapy, deep vein thrombosis

## Abstract

Pulmonary embolism (PE) is a life-threatening condition resulting from the obstruction of pulmonary arteries by blood clots, usually originating from deep veins. Symptoms of PE might vary from nothing to sudden death. Clinically, individuals may present very differently. When a diagnosis of PE is suspected, any possible life-saving intervention must be implemented because survival from cardiac arrest following PE is often quite low. Although there are not many randomized controlled trials that provide guidelines for treating suspected PE in cardiac arrest victims, the few published case reports and other minor studies suggest that thrombolysis and other therapies are associated with good outcomes. We report a patient with PE who presented in cardiac arrest with its clinical, electrographic, and radiologic findings, along with the appropriate therapy chosen based on hemodynamic stability. It is important to intervene early to prevent severe complications and improve the patient’s outcomes.

## Introduction

A pulmonary embolism (PE) is a life-threatening condition, with highly variable and non-specific presentations [1-3]. When clotted blood enters the pulmonary artery circulation, it can cause PE. Deep vein thrombosis (DVT) is the most common cause of PE. PE has a case fatality rate of 45% when combined with circulatory shock, while only 4% to 5% of patients with PE have shock. The case fatality rate depends on age, concomitant diseases, and the dynamic severity of the PE. The case fatality rate for individuals under 50 years of age with hemodynamically stable PE who do not have any other comorbidities is 1% [4]. The mortality rate for massive PE is 30% [5], and it may reach 95% in cases where PE results in cardiac arrest [6]. PE with cardiac arrest or hemodynamic instability should be diagnosed and treated as early as possible for better outcomes.

## Case presentation

**Figure 1:**
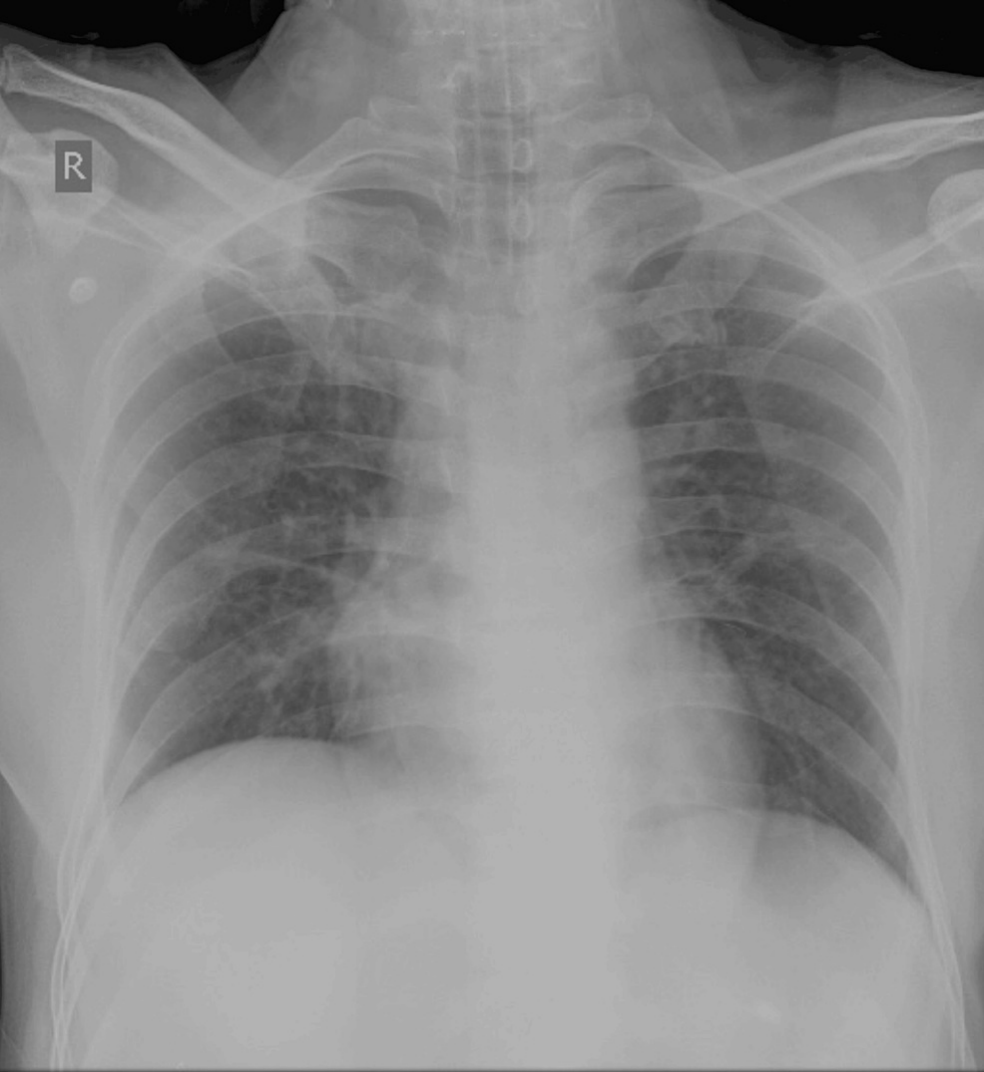
Chest radiography showing no significant abnormality and an endotracheal tube in situ.

**Figure 2:**
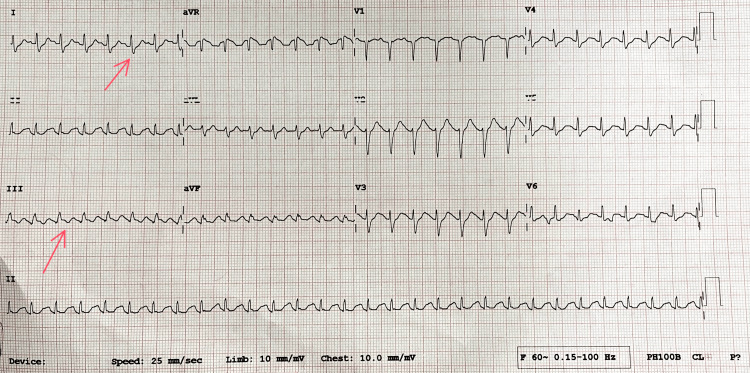
Electrocardiography suggestive of S1T3 with sinus tachycardia.

**Figure 3:**
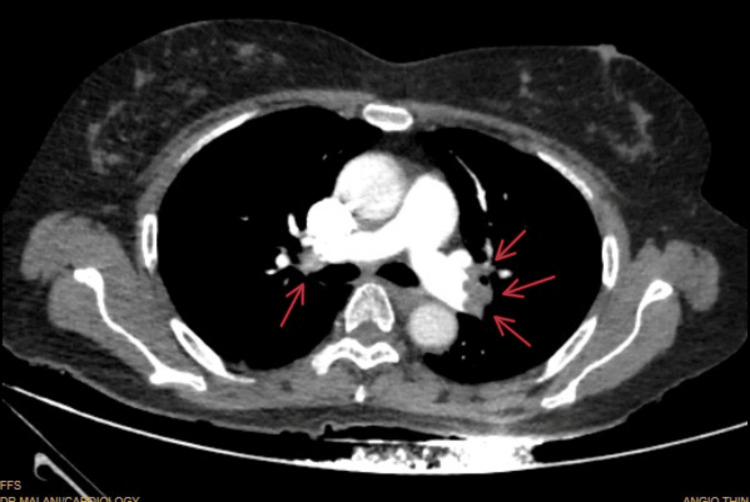
Computed tomography pulmonary angiography suggestive of bilateral pulmonary embolism.

A 32-year-old male came to the emergency in an unresponsive state with gasping respiration. Pulse rate and blood pressure were not recordable. The monitor showed organized cardiac activity at 32/minute and rhythm identified as pulseless electrical activity (PEA). Immediately, cardiopulmonary resuscitation (CPR) was started according to standard advanced cardiac life support protocol. Return of spontaneous circulation was achieved after five cycles of CPR. The advanced airway was secured during ongoing CPR. Post return of spontaneous circulation pulse rate and blood pressure were 170 beats/minute and 50/30 mmHg, respectively. Vasopressor support was started accordingly. Respiratory rate and oxygen saturation were 35 breaths/minute and 75%, respectively, on 100% FiO_2_ and positive end-expiratory pressure of 5 cmH_2_O. On auscultation, the chest was clear with the chest radiograph showing no significant abnormality and endotracheal tube in situ (Figure 1). Electrocardiography (ECG) showed S1T3 with sinus tachycardia (Figure 2). Point-of-care ultrasound (POCUS) was suggestive of the right atrium and right ventricle dilation, positive Mc Connell’s sign, and a clot in the right atrium (Video 1). DVT screening was positive for the patient. Arterial blood gas analysis was suggestive of metabolic and respiratory acidosis. Before arrival, the patient gave a history of breathlessness and chest pain for two hours. The patient was a chronic smoker and alcoholic. The Well’s score was 7.5. The patient was thrombolised with tenecteplase 30 mg intravenous bolus dose. Following this, the patient was started on low-molecular-weight heparin (LMWH). Vasopressor requirement decreased, and oxygenation and Glasgow Coma Scale (GCS) score improved significantly. Six hours later, the patient was off vasopressor and oxygen support with significant improvement in the GCS score. The patient was extubated and shifted to the intensive care unit. Computed tomography pulmonary angiography (CTPA) was done after stabilization, suggestive of bilateral PE (Figure 3). The patient was discharged with good neurological outcomes.

**Video 1:** Point-of-care ultrasound suggestive of a mobile clot in the right atrium along with a right atrium and right ventricle dilation.

## Discussion

A cardiac arrest is a sudden collapse that is followed by unconsciousness, no spontaneous breathing, and a loss of central pulse. The only interventions that have been linked to better outcomes during cardiac arrest are the prompt initiation of high-quality CPR, the use of an external defibrillator, and the identification and treatment of reversible causes of cardiac arrest [7].

Hypoxia, hypovolemia, hyperkalemia or hypokalemia, hydrogen ion (acidosis), hyperthermia or hypothermia, tension pneumothorax, thrombosis (pulmonary), tamponade (cardiac), thrombosis (coronary), and toxins (5H’s and 5T’s) are the reversible causes of cardiac arrest. It is necessary to suspect PE in individuals who have undergone cardiac arrest [7,8].

According to Kürkciyan et al., PE accounted for 4.8% of all cardiac arrests, where PEA was identified as the most common (63%) initial cardiac rhythm followed by asystole (32%) and ventricular fibrillation (5%) [9].

Occlusion of the pulmonary artery by the larger clot will result in raised pulmonary artery pressure, ultimately leading to right ventricular dilation and strain. It will also cause myocardial damage with the release of B-type natriuretic peptide and troponin. Increased right ventricle to left ventricular ratios on computed tomography scans or echocardiograms (right ventricular dilatation or injury), elevated B-type natriuretic peptides or troponin (right ventricular strain), and signs of acute pulmonary hypertension on 12-lead ECG indicate an increased risk of circulatory shock, right heart failure, and death [10-12]. One of the two pathways usually accounts for the death of patients with PE: (1) sudden, nearly complete blockage of the pulmonary arteries, resulting in asystole or PEA due to an ischemic impact on the His-Purkinje conduction pathway; (2) circulatory shock and progressive right heart failure that occurs over hours to days.

Patients with PE may have variable clinical presentations. Dyspnea that is not explained by auscultatory signs, ECG abnormalities, or a chest radiograph with no obvious alternative diagnosis will increase suspicion of PE. Regarding symptoms, the second most prevalent one is chest pain with pleuritic characteristics; however, more than 50% of patients diagnosed with PE in the emergency department do not have chest pain [4]. Abnormal vital signs suggestive of PE are tachycardia, tachypnea, hypoxia, hypotension, and sometimes mild fever. About 3% to 4% of emergency department patients with PE experience syncope, and an additional 1% to 2% report having disorientation or a new-onset “seizure” [13].

Risk factors associated with PE are age (>50 years), obesity, pregnancy and postpartum state, prior PE, DVT, malignancies, thrombophilias, immobility, recent surgery or trauma bed rest, indwelling catheters, long-distance travel (>6 hours), congestive heart failure, smoking, stroke, non-infectious inflammatory conditions such as inflammatory bowel diseases, lupus, and nephrotic syndrome. Here, the patient had smoking and DVT as risk factors.

Non-specific findings of PE on chest radiographs include cardiomegaly, basilar atelectasis, infiltrates, or pleural effusion. About 5% of patients have a wedge-shaped patch of lung oligemia (called Westermark’s sign), usually from entire lobar artery occlusion, or a peripheral dome-shaped dense opacification (Hampton’s hump), suggesting pulmonary infarction. Suspicion of PE should be kept if there is dyspnea or hypoxemia with clear lung on physical examination and chest radiography.

The likelihood of PE increases if an ECG shows signs of acute pulmonary hypertension, including pulse rate >100 beats/minute, T-wave inversion in leads V1 to V4, complete or incomplete right bundle branch block, and the S1-Q3-T3 pattern [14]. In this case, the patient had ECG findings of S1T3 with sinus tachycardia.

The risk stratification of suspected PE can be done by clinical gestalt and various scoring systems such as Well’s score, revised or simplified Geneva score, and PE rule-out criteria.

CTPA is the gold standard for the diagnosis of pulmonary thromboembolism. However, POCUS is an effective tool for early diagnosis and treatment in hemodynamically unstable or cardiac arrest patients. POCUS findings suggestive of PE include DVT, right heart thrombi, increased pulmonary artery pressure, right ventricular strain and dilation, tricuspid regurgitation, right ventricular systolic dysfunction, positive McConnell’s sign, and bowing of interventricular septum. McConnell’s sign is the most specific for PE, defined as right ventricular free wall akinesis with sparing of the apex [15].

The treatment approach for PE depends on its severity. PE can be classified as massive, submassive, and less severe PE based on severity. Patients with massive PE have a systolic blood pressure (SBP) of less than 90 mmHg, a decline in baseline SBP of more than 40%, or an SBP of less than 100 mmHg with a history of hypertension. Patients with submassive PE have normal or near-normal blood pressure but with evidence of cardiopulmonary stress. All other patients are classified as less severe PE. Treatment options for low-risk PEs (less severe PEs) are unfractionated heparin or LMWH and can also include oral apixaban or rivaroxaban [16]. Systemic fibrinolysis should be considered in patients with massive PE and more severe submassive PE after ruling out all contraindications and any of the following: cardiac arrest; respiratory failure (oxygen saturation of less than 90%) despite oxygen supplementation; hypotension (SBP less than 90 mmHg); or evidence of right heart strain on echocardiography or elevated levels of troponin and B-type natriuretic peptides [17]. Alteplase is the only Food and Drug Administration-approved drug for systemic fibrinolysis in the case of PE. However, success with other thrombolytic agents (tenecteplase) has been described in one study [18]. Scholz et al. noted that seven out of 17 (41%) patients who received thrombolytic treatment during cardiac arrest after PE had favorable outcomes [19], and Hopf et al. showed similar results in five out of six patients [20]. The American Heart Association and the European Resuscitation Council have recommended the use of thrombolytic treatment when PE is either identified or presumed to be the cause of cardiopulmonary arrest, despite the evidence being weak [8,17]. Mechanical thrombectomy, extracorporeal membrane oxygenation, and surgical embolectomy are other treatment options PE during cardiac arrest [8,17].

## Conclusions

As mortality is very high in patients with cardiac arrest following PE, early detection and intervention are necessary to prevent worse outcomes. POCUS is a crucial screening tool to diagnose such cases. Promising outcomes have been observed with several thrombolytic drugs and other therapy techniques in the few published case reports and other small research on this subject. Nevertheless, there are not enough randomized controlled trials to recommend a course of action for treating a cardiac arrest victim with suspected PE. A well-designed multicenter randomized controlled trial is needed to resolve current uncertainties.

## References

[1] Robin ED (1977). Overdiagnosis and overtreatment of pulmonary embolism: the emperor may have no clothes. Ann Intern Med.

[2] Hampson NB (1995). Pulmonary embolism: difficulties in the clinical diagnosis. Semin Respir Infect.

[3] Rizkallah J, Man SF, Sin DD (2009). Prevalence of pulmonary embolism in acute exacerbations of COPD: a systematic review and metaanalysis. Chest.

[4] Pollack CV, Schreiber D, Goldhaber SZ (2011). Clinical characteristics, management, and outcomes of patients diagnosed with acute pulmonary embolism in the emergency department: initial report of EMPEROR (Multicenter Emergency Medicine Pulmonary Embolism in the Real World Registry). J Am Coll Cardiol.

[5] Stulz P, Schläpfer R, Feer R, Habicht J, Grädel E (1994). Decision making in the surgical treatment of massive pulmonary embolism. Eur J Cardiothorac Surg.

[6] Bailén MR, Cuadra JA, Aguayo De Hoyos E (2001). Thrombolysis during cardiopulmonary resuscitation in fulminant pulmonary embolism: a review. Crit Care Med.

[7] Callaway CW, Soar J, Aibiki M (2015). Part 4: Advanced Life Support: 2015 International Consensus on Cardiopulmonary Resuscitation and Emergency Cardiovascular Care Science With Treatment Recommendations. Circulation.

[8] Lott C, Truhlář A, Alfonzo A (2021). European Resuscitation Council Guidelines 2021: Cardiac arrest in special circumstances. Resuscitation.

[9] Kürkciyan I, Meron G, Sterz F (2000). Pulmonary embolism as a cause of cardiac arrest: presentation and outcome. Arch Intern Med.

[10] Aujesky D, Obrosky DS, Stone RA (2005). Derivation and validation of a prognostic model for pulmonary embolism. Am J Respir Crit Care Med.

[11] Shopp JD, Stewart LK, Emmett TW, Kline JA (2015). Findings from 12-lead electrocardiography that predict circulatory shock from pulmonary embolism: systematic review and meta-analysis. Acad Emerg Med.

[12] Ouriel K, Ouriel RL, Lim YJ, Piazza G, Goldhaber SZ (2017). Computed tomography angiography with pulmonary artery thrombus burden and right-to-left ventricular diameter ratio after pulmonary embolism. Vascular.

[13] Carpenter SL, Richardson T, Hall M (2018). Increasing rate of pulmonary embolism diagnosed in hospitalized children in the United States from 2001 to 2014. Blood Adv.

[14] Marchick MR, Courtney DM, Kabrhel C (2010). 12-lead ECG findings of pulmonary hypertension occur more frequently in emergency department patients with pulmonary embolism than in patients without pulmonary embolism. Ann Emerg Med.

[15] McConnell MV, Solomon SD, Rayan ME, Come PC, Goldhaber SZ, Lee RT (1996). Regional right ventricular dysfunction detected by echocardiography in acute pulmonary embolism. Am J Cardiol.

[16] Kearon C, Akl EA, Comerota AJ (2012). Antithrombotic therapy for VTE disease: Antithrombotic Therapy and Prevention of Thrombosis, 9th ed: American College of Chest Physicians Evidence-Based Clinical Practice Guidelines. Chest.

[17] Lavonas EJ, Drennan IR, Gabrielli A (2015). Part 10: Special Circumstances of Resuscitation: 2015 American Heart Association Guidelines Update for Cardiopulmonary Resuscitation and Emergency Cardiovascular Care. Circulation.

[18] Hefer DV, Munir A, Khouli H (2007). Low-dose tenecteplase during cardiopulmonary resuscitation due to massive pulmonary embolism: a case report and review of previously reported cases. Blood Coagul Fibrinolysis.

[19] Scholz KH, Hilmer T, Schuster S, Wojcik J, Kreuzer H, Tebbe U (1990). [Thrombolysis in resuscitated patients with pulmonary embolism]. Dtsch Med Wochenschr.

[20] Hopf HB, Flossdorf T, Breulmann M (1991). [Recombinant tissue-type plasminogen activator for the emergency treatment of perioperative life-threatening pulmonary embolism (stage IV). Results in 7 patients]. Anaesthesist.

